# Removal of Contaminants of Emerging Concern from Wastewater Using Photocatalytic Membranes: Current Status and Challenges

**DOI:** 10.3390/membranes16040153

**Published:** 2026-04-21

**Authors:** Nelson Kipchumba, Innocentia G. Mkhize, Benton Otieno, Hilary L. Rutto, Seteno K. Ntwampe

**Affiliations:** 1 Chemical Engineering Department, Faculty of Engineering and the Built Environment, Durban University of Technology, Durban 4000, South Africa; setenon@dut.ac.za; 2 Water Sanitation and Hygiene Research & Development (WASH R&D) Centre, University of KwaZulu-Natal, Durban 4001, South Africa; bentononyango@gmail.com; 3 School of Chemical and Metallurgical Engineering, University of Witwatersrand, Johannesburg 2000, South Africa; hilary.rutto@wits.ac.za

**Keywords:** photocatalytic membrane, wastewater, contaminants of emerging concern, synthesis, scale-up

## Abstract

The increasing presence of contaminants of emerging concern (CECs) in surface and groundwater is a global concern due to their toxicity, persistence, and bioaccumulation, which lead to undesired effects. Conventional wastewater treatment processes are unable to remove these CECs, necessitating advanced treatment strategies to remove them effectively. Among advanced strategies, photocatalytic membrane treatment has attracted considerable interest among researchers. This review critically examines the fundamental principles governing the performance of photocatalytic membranes. It identifies significant challenges, including photocatalyst leaching, light accessibility, intermediates’ toxicity, and scalability of synthesis and immobilisation techniques. It explains why these factors significantly hinder long-term stability, scalability, and practical deployment of photocatalytic membrane systems and provides potential solutions. Through gap analysis, the review has identified rigorous techno-economic analysis, real-world wastewater validation, and systematic toxicity assessment of degradation intermediates as areas of further study. These targeted actions provide clear pathways to enhance the viability, safety, and commercial readiness of photocatalytic membrane systems.

## 1. Introduction

Contaminants of emerging concern (CECs) are newly identified natural or synthetic chemicals detected in wastewater, surface, and groundwater, whose adverse effects are still being determined and that have raised significant challenges due to properties such as persistence, toxicity, and bioaccumulation [1]. The CECs can be classified by source. They originate from pharmaceuticals, hormones, personal care products, plasticisers, fire retardants and surfactants, pesticides, and dyes [2]. The relative contributions of these sources to the CECs vary considerably. Pharmaceuticals and personal care products account for 35–45% of detected CECs in municipal wastewater effluent, followed by pesticides (20–30%) and industrial chemicals such as plasticisers and surfactants (15–25%), according to previous studies [3,4]. The reported concentrations of individual CECs in raw municipal wastewater typically range from 10 to 10,000 ng/L. In contrast, concentrations in treated water often remain above 100 ng/L, highlighting the inefficiency of conventional activated sludge treatment systems used by municipal wastewater treatment plants (WWTPs) to remove CECs [5]. A previous study reported typical effluent concentrations of up to 40% for various CECs [6]. Their low concentrations (ng/L to µg/L) are below the operational sensitivity of biological systems, meaning that activated sludge microorganisms do not metabolise them effectively [7]. As a result, these contaminants bypass traditional processes and are discharged into receiving water bodies [8].

Conventional WWTPs are designed to remove simple organic matter and nutrients but not specifically CECs. This limitation arises because WWTPs primarily rely on primary sedimentation, biological oxidation, and secondary clarification processes that target biodegradable organic compounds and suspended solids [9]. Most CECs, such as antibiotics, stimulants, and industrial chemicals, have complex molecular structures, high stability, and low biodegradability, which allow them to resist microbial degradation and physical adsorption in conventional treatment stages [10].

The presence of CECs causes adverse effects, such as endocrine disruption, in aquatic environments, wildlife, and humans, even at low environmental concentrations. Antibiotics such as trimethoprim and sulfamethoxazole, classified as CECs, have been detected in surface waters at concentrations ranging from 1 to 19,190 ng/L. Antibiotic concentrations below the effective dosage lead to the proliferation of antibiotic-resistant genes (ARGs) in aquatic microorganisms [11]. These ARGs can be transferred to pathogenic bacteria, posing a direct risk to public health by reducing the efficacy of existing antibiotics [11,12]. Endocrine-disrupting compounds (EDCs) such as bisphenol A, 17α-ethinylestradiol, and nonylphenol have been detected in rivers and lakes at concentrations as low as 4 ng/L. Yet, they can induce feminisation in male fish, alter sex ratios, and impair reproductive development in aquatic fauna [13,14]. In humans, CECs have been identified, attributed to, and associated with health complications, such as diseases and inflammatory conditions [1].

The limitations of conventional treatment methods have highlighted the need to develop and implement advanced and more efficient treatment technologies to improve the quality of final effluent from WWTPs, thereby ensuring the safety of water in the environment and for human use. The unknown effects of cocktailing or the synergistic effects of multiple CECs emphasise the importance of removing these contaminants [15]. Among existing advanced treatment techniques, photocatalysis, an advanced oxidation process, and membrane filtration have been widely applied for the removal of CECs [16]. Through photocatalysis, the CECs can be mineralised to H_2_O and CO_2_, while membrane filtration prevents the passage of a wide range of CECs. Titanium dioxide (TiO_2_) is the most widely used nanosized photocatalyst because it is inert, less toxic, widely available and has a high surface area [17]. The exposure of photocatalysts to ultraviolet (UV) or visible light generates electron–hole pairs, which are utilised to drive redox reactions that eventually oxidise the CEC molecules into simple products, primarily carbon dioxide and water [18]. However, photocatalysis with TiO_2_ requires high-energy input (ultraviolet light) and may not fully mineralise CECs, as stable intermediates form. Membrane filtration utilises different pore sized filters such as micro filters (pore size 100–2000 nm, 5–500 kPa), ultra filters (2–100 nm pore size, less than 1 MPa), nano filters (1–10 nm pore size, less than 4 MPa) and reverse osmosis filters (less than 2 nm pore size and 5–10 MPa) to separate contaminants from water by pore size exclusion and stearic hinderance [19,20]. However, despite the potential, membranes are prone to fouling [21,22]. Systems that integrate photocatalysis and membrane filtration, i.e., photocatalytic membranes, have been developed to overcome the shortcomings of the individual processes [23]. The functional membrane part separates and concentrates CECs at the photocatalyst surface, enabling their effective degradation into simpler products and reducing membrane fouling [24].

Extensive research has led to a surge in the field of photocatalytic membrane performance optimisation for the elimination of CECs. The efficiency of filtration, the degradation process, and long-term stability have been explored using various photocatalyst materials, membrane and reactor designs, and operating conditions [25,26,27,28,29]. However, some challenges remain to be addressed, including catalyst deactivation, leaching, persistent membrane fouling, high pumping energy requirements, and the need for efficient visible-light-based systems [30].

Past review studies have focused on materials characterisation and the performance of photocatalytic membranes. They have given limited attention to identifying and discussing challenges such as the detachment of photocatalysts, light accessibility, and unscalable fabrication techniques that arise from integrating photocatalysis and membrane filtration, which have resulted in the hindrance of scaling up photocatalytic membranes [31,32,33,34,35,36,37]. This review critically evaluates the current state of photocatalytic membrane (PM) technology for the removal of CECs from wastewater and assesses its technological readiness, including scale-up requirements. It reviews and provides detailed potential solutions to the challenges of integrating photocatalysis with membrane filtration, including catalyst leaching, light accessibility, the toxicity of reaction intermediates, and the scalability of synthesis and immobilisation techniques that are often overlooked [32,38,39,40]. This will provide a strong basis for guiding the scaling up of photocatalytic membranes.

## 2. Contaminants of Emerging Concern: Existence, Identification, and Effects

CECs are among the most important classes of organic contaminants. These chemicals exhibit harmful effects such as endocrine disruptions at concentrations even below toxic levels [41]. They enter the aquatic environment and water circulation systems through multiple pathways, including human and animal excreta, improper disposal of unused drugs and plastics, emissions from manufacturing processes, agricultural runoff from pesticide use, and leachates from landfills [41]. The reason for their environmental mobility is that CECs incorporated into everyday commodities are often weakly bound to matrices via non-covalent interactions, allowing them to leach easily once the product degrades or is discarded [42].

Hospitals, clinics, and the pharmaceutical industry contribute significantly to CEC loading in wastewater. They discharge effluents containing high levels of pharmaceuticals, including antibiotics, anti-inflammatory agents, and synthetic hormones used for various medical treatments [43]. Studies have reported hospital effluent concentrations of pharmaceuticals ranging from 100 ng/L to more than 50,000 ng/L, which are orders of magnitude higher than those found in domestic wastewater [44]. In addition to direct discharge, the incomplete metabolism of administered drugs in humans and animals results in unmetabolised fractions being excreted into sewage systems [44]. The agricultural sector also plays a substantial role in CEC contamination through the widespread application of insecticides, antibiotics, fungicides, and growth regulators in both crop protection and livestock management [45,46]. Runoff and infiltration from agricultural lands transport these chemicals to surface and groundwater reservoirs, where they persist for extended periods due to their chemical stability [47]. CECs are not easily degradable due to their complexity and long carbon chains, which make them stable and resistant to biodegradation, as indicated by their high log K_OW_ values, more than 2 [48]. Notably, a previous study has established that log K_OW_ values of 4.5 or higher better describe bioaccumulation in animals and the environment [49]. Furthermore, the high log K_OW_ values of CECs enhance their partitioning into sediments or extracellular polymeric substances (EPSs) [50]. An EPS acts as a sticky multifunctional matrix that binds CECs through adsorption, complexation, and hydrophobic interactions, thereby reducing their mobility in water and promoting their accumulation. Combinatorially, with other CECs such as microplastics, the effects of sediment partitioning are further enhanced. Partial or incomplete degradation of CECs within these systems can even generate more toxic transformation products through oxidation, hydrolysis, or the splitting of functional groups [51,52]. For example, deconjugation of steroid hormones during secondary treatment regenerates biologically active hormones, thereby increasing the endocrine-disrupting potential of wastewater effluents [53].

Recent advances in detection technologies have expanded the understanding of CECs’ diversity and distribution. High-resolution analytical methods, such as gas chromatography–mass spectrometry (GC-MS) and high-performance liquid chromatography–mass spectrometry (HPLC-MS), and biological screening assays have identified thousands of compounds with endocrine-disrupting potential [53]. These methods now permit quantification of CECs at sub-nanogram levels, revealing their widespread presence even in treated water.

The persistence of these chemicals has profound ecological and health consequences. Low concentrations of antibiotics (below minimum inhibitory concentrations) provide selective pressure that promotes the development of antibiotic-resistant bacteria and facilitates the proliferation of ARGs within microbial communities [54]. Such resistance can spread through gene transfer, posing a global threat to public health. Numerous studies have demonstrated that anti-inflammatory drugs, pesticides, surfactants, and industrial chemicals exhibit endocrine-disrupting properties, interfering with hormonal balance and reproduction in wildlife [55,56,57].

The ecological manifestations of endocrine disruption are diverse and severe: feminisation of male fish, intersex development, gonadal underdevelopment, reduced fertility, weakened eggshells in birds, and population declines in sensitive aquatic species [55,56]. In terrestrial and human systems, exposure to endocrine-disrupting chemicals has been correlated with metabolic disorders such as type 2 diabetes and obesity, reproductive complications including infertility and abnormal pregnancies, disruption of developmental stages, and various hormone-related cancers [58]. These effects highlight the urgent need to improve the management and removal of CECs from environmental matrices. To better illustrate the diversity of these contaminants, Table 1 summarises selected classes of CECs, representative compounds, and their reported ecological or biological effects.

## 3. Fundamentals of Photocatalytic Membranes

The current WWTPs primarily employ biological and physicochemical processes to treat wastewater containing CECs before discharge into water bodies. Biological and physicochemical methods are ineffective in removing CECs due to their low concentration and recalcitrance [69]. CECs eventually enter water bodies. This, therefore, necessitates the use of advanced treatment technologies, including membrane filtration, advanced oxidation processes (AOPs), such as photocatalysis, and hybrid systems, such as photocatalytic membranes, to eliminate CECs from wastewater. Table 2 shows the total organic carbon (TOC) removal achieved by various AOP-based methods in the treatment of a representative CEC. Experimental evaluations reveal that hybrid photocatalytic membranes can achieve 95–98% removal of model pollutants like carbamazepine and ibuprofen, compared with less than 70% for photocatalysis or filtration alone [70,71,72].

### 3.1. Photocatalytic Membrane Fabrication

The main aim of recent fabrication innovations is to balance membrane permeability, photocatalyst stability, and active-site accessibility while ensuring scalability and cost-effectiveness [76]. However, many reported fabrication strategies still prioritise laboratory-scale performance metrics over long-term operational robustness and cost–benefit considerations. Thus, fabrication routes must be evaluated not only for their efficiency in embedding or exposing photocatalysts but also for their influence on mechanical integrity, fouling resistance, and recyclability under real treatment conditions [31]. Photocatalytic membranes have been fabricated using various techniques that impart characteristic performance, such as antifouling capability and pollutant degradation, by exposing photocatalytic active sites [77]. The fabrication techniques can be physical or chemical and include phase inversion, chemical grafting, in situ synthesis, sol–gel, pressure deposition, physical and electric atomisation, electrospinning, and layer-by-layer (LbL) assembly [78]. While this diversity of techniques enables tailored membrane design, it also complicates standardisation and scale-up, as many methods rely on controlled laboratory conditions, specialised equipment, or multi-step processing that may not be compatible with industrial membrane manufacturing lines [76].

Recent work emphasises hybridising these techniques, such as combining phase inversion with surface grafting, which can yield membranes with hierarchical porosity and superior photocatalytic dispersion, thereby bridging the trade-off between permeability and photoactivity [79,80,81]. However, such hybridisation often introduces additional complexity, higher fabrication costs, and potential reproducibility challenges. Phase inversion and chemical grafting are preferred for their simplicity and cost-effectiveness; they are limited by photocatalyst leaching and partial pore blockage [82]. Table 3 lists the grafting techniques and the features they impart to the photocatalytic membrane.

Techniques such as electrospinning and LbL assembly, though they provide high surface reactivity, face scalability and durability challenges [89,90]. Hybrid strategies that integrate multiple methods of phase inversion and in situ growth or spray-and-sol–gel, have emerged as promising pathways to high-performance long-life membranes for continuous-flow water treatment [91]. Despite these advantages, their industrial translation remains constrained by scalability, mechanical durability, and cost. Future directions will involve integrating 2D materials, metal–organic frameworks (MOFs), and carbon quantum dots via scalable fabrication, coupled with digital-twin-based design simulations for performance prediction [92,93]. Innovative fabrication should prioritise sustainable solvents, low-temperature synthesis, and recyclable composites to align with green chemistry principles, thereby ensuring the practical viability of photocatalytic membranes in circular water systems [94]. Despite advancements in materials and the synthesis of photocatalytic membranes, integration challenges still hinder their full potential [95].

### 3.2. Challenges of Integrated Photocatalysis and Membrane Filtration and Solutions

Integrating photocatalysis and membrane technologies enhances both approaches by addressing their individual limitations, such as photocatalyst reusability and membrane fouling. Despite improvements at the material and fabrication levels, the synergistic integration still presents several operational and material challenges. Among the most critical are catalyst leaching, limited light accessibility, the toxicity of reaction intermediates, and the scalability of synthesis and immobilisation techniques [78,96,97].

#### 3.2.1. Permeability

Catalyst loading plays a critical role in determining both membrane permeability and light accessibility in photocatalytic membrane systems. Increasing photocatalyst loading generally enhances the availability of photoactive sites for CECs degradation; however, excessive loading can significantly reduce the membrane permeability due to pore blockage, increased surface roughness, and the formation of thicker catalytic layers that restrict water flow, thereby increasing the hydraulic resistance and pumping energy [24]. Additionally, optimal catalyst loading improves photon absorption and photocatalytic efficiency; however, beyond a certain threshold, agglomeration and layer thickening cause light scattering and shielding, which limit photon penetration into deeper catalyst layers. This results in the underutilisation of immobilised photocatalyst particles and decreased light utilisation efficiency [98]. The largest challenge lies in balancing photocatalyst stability and activity without compromising membrane permeability or light transmission, a trade-off that continues to limit large-scale practical applications [76]. Table 4 presents studies reporting optimal photocatalyst loadings and associated permeability limits.

#### 3.2.2. Catalyst Leaching

Catalyst leaching from the membrane hinders the stable long-term operation and reduces the permeability through pore blockage, loss of self-cleaning efficiency, reduced hydrophilicity and irreversible fouling [102,103]. Leaching is primarily caused by mechanical stress [104], chemical degradation [105], and inherent weaknesses in immobilisation design [106]. Mechanical stress arises from shear forces and turbulence during filtration and backwashing, which detach immobilised photocatalysts [107]. In slurry-type photocatalytic systems, nanoparticles abrade the membrane surface, accelerating both structural fatigue and photocatalyst detachment [108]. UV light and reactive oxygen species (ROS) also induce the nonselective degradation of polymeric matrices [109], leading to detachment. A study observed an 18% loss of the initially immobilised photocatalyst from a PVDF membrane after 250 h of cross-flow (pH 7–8), quantified by ICP-MS of permeate plus mass balance on the membrane [110], while another reported an 11% loss from TiO_2_–PTFE membranes [111]. Under acidic or basic wastewater conditions, dissolution rates of metal oxide catalysts such as Fe_2_O_3_ or ZnO can increase by 30–50%, particularly at a pH below 4 or above 9, as the dissolution of metal ions is thermodynamically favoured [112,113]. A previous study detected dissolved photocatalysts up to 97,000 ng/L, while )another study identified iron ions in the filtrate after reuse cycles, confirming the chemical instability [114,115]. Leaching not only diminishes the photocatalytic efficiency but also poses a risk of secondary contamination as detached particles enter the permeate stream [78]. The presence of nanosized photocatalysts, such as titanium dioxide, in the environment poses a threat of genotoxicity, inflammation, oxidative stress, and compromised bioreactions when nanoparticles interact with other free radicals [116,117]. To mitigate leaching, multiple strategies to improve immobilisation are proposed. Strengthening attachment through chemical anchoring, crosslinking, and surface functionalisation has proven effective [118]. This involves pre-treating the membrane with oxygen plasma, UV, or ozone to introduce –OH or –COOH functional groups, followed by graft polymerisation with monomers such as acrylic acid or glycidyl methacrylate. These groups form covalent bonds with metal oxides or MOFs photocatalysts via coordination or esterification reactions, thereby enhancing the attachment strength [119,120,121,122]. Additionally, intermediate coupling layers such as polydopamine or silane linkers are increasingly used to promote adhesion between inorganic catalysts and polymeric membranes [123,124,125]. Studies should therefore include quantitative leaching measurements (e.g., metal–ion concentrations in permeate), multi-cycle or long-term operational data, and post-operation characterisation to assess structural or chemical changes.

#### 3.2.3. Light Accessibility

Light accessibility determines the extent of photocatalytic activation on or within the membrane structure, because light must pass through the wastewater matrix to reach the photocatalyst. Turbidity and dissolved natural organic matter (NOM) significantly reduce the irradiance. A study demonstrated that tetracycline degradation efficiency decreased by 42% when the solution turbidity increased from 10 to 60 NTU [126]. Similarly, another study reported that increased opacity in coated photocatalytic membranes hindered photoactivation [127], while another study found a decline in degradation beyond a 3% PVA coating thickness [99]. To enhance light accessibility, several design strategies have emerged. Increasing the specific surface area and optical transmittance of membranes by synthesising ultrathin, porous, and semi-transparent films enables higher light penetration. Transparent polymer–oxide nanocomposites have shown higher photocatalytic activity after 180 min of light irradiation, with contaminant mineralisation exceeding 70% [128]. Practical implementation can involve reducing the thickness of the wastewater layer above the membrane, employing side-illumination photoreactors, or embedding optical fibres or photonic structures to more uniformly distribute light across the membrane surface [129,130,131]. However, thinning and porosity increase may weaken the membrane mechanically, necessitating support layers or reinforcing meshes to maintain stability. Membrane fouling blocks pores and reduces transparency [132]. Although photocatalytic self-cleaning partially mitigates fouling, real wastewater systems often require chemical cleaning, periodic backwashing, or low-frequency ultrasound cleaning to restore flux and photon access [133]. A previous study investigated the variation in light absorption with photoreactor geometry. Optimal light absorption was observed at 75% of the photoreactor diameter and could serve as a basis for the optimal placement of immobilised photocatalytic membranes in the reactor [134]. In a photoreactor, light accessibility is often reduced by fouling on lamps or reactor walls, leading to uneven photodegradation [135]. Solutions include automated wiping of light barriers, strategic mapping of dark zones, and installation of reflective surfaces or light concentrators to focus radiation on membranes [136]. Integration of sunlight with UV lamps reduces electricity consumption and enhances 24 h operation [137]. To enable energy-based comparisons, studies should report the electrical energy per order or related energy efficiency metrics, particularly for UV-driven systems [138]. Additionally, control experiments (dark filtration, light without a catalyst, and membrane without a catalyst) are essential and should be documented [139].

#### 3.2.4. Toxicity

The toxicity of the degradation byproducts affects the environment and public health. It results from the discharge of incomplete degraded contaminants that have been subdivided from the parent emerging contaminant, thereby increasing the number of contaminants [140]. This raises significant environmental and public health concerns, particularly where treated water is discharged into receiving water bodies or reused without a comprehensive toxicity assessment. In some instances, the degradation byproducts are more toxic than the parent molecule, as shown by bioassays. For example, the photocatalytic degradation of parabens yields monohydroxylated parabens, which are more toxic and less characterised in aquatic environments [141]. Additionally, increased toxicity has been observed with other CECs such as bezafibrate, ibuprofen, diclofenac, and carbamazepine [142]. Failure to account for this transformation undermines the environmental credibility of photocatalytic membrane technology and may lead to unintended ecological consequences. This can be addressed by monitoring degradation byproducts to determine the required increase in hydraulic retention time (HRT), thereby enabling larger degradation durations. Additionally, the photocatalytic membrane wastewater treatment can be made continuous with a retentate recycle stream to minimise toxic volume discharge, as the retentate phase is a volume stream composed of highly concentrated reaction intermediates that can be more toxic [143]. This will allow for extended degradation duration and minimise secondary environmental contamination. The photocatalytic membrane performance should not be reported solely as the percentage removal. Minimum standards include time-resolved concentration profiles, apparent kinetic constants (e.g., pseudo-first-order rate constants), and precise identification of target compounds and initial concentrations [144]. Where relevant, transformation products and mineralisation indicators (e.g., TOC removal) should be reported to distinguish between partial oxidation and complete degradation [145]. Toxicity assays, including acute and chronic ecotoxicity tests, should be incorporated to verify that the treatment reduces the overall toxicity rather than redistributing it among transformation products [146].

#### 3.2.5. Scalability of Synthesis and Immobilisation Techniques

The scalability of synthesis and immobilisation techniques remains a bottleneck. Laboratory-scale methods, such as dip coating or batch sol–gel processing, are simple and suited to lab-scale membranes, but they lack uniformity and are difficult to control on industrial-scale membranes [147]. Scaling requires more consistent continuous processes, such as spray coating, chemical vapour deposition (CVD), or roll-to-roll layer assembly, to ensure uniform catalyst distribution as variations in coating thickness, photocatalyst dispersion, and adhesion across larger membrane areas can lead to uneven photoactivity, localised fouling, and accelerated catalyst detachment, ultimately undermining long-term operational reliability [148]. In recent pilot studies, automated spray systems achieved over 90% uniform coverage on membranes up to 1 m^2^, demonstrating industrial feasibility [149]. To ensure durability, these scalable coatings are often crosslinked using low-energy UV curing or annealed at moderate temperatures (100–150 °C) to form stable bonds between the photocatalyst and membrane support. While these approaches can enhance short-term adhesion, their long-term effectiveness under continuous irradiation, backwashing, and chemical exposure remains insufficiently validated. Thermal post-treatment may also alter the membrane pore structure, polymer crystallinity, or mechanical properties, potentially compromising permeability or flexibility [150]. Furthermore, UV curing and annealing steps increase energy demand and process complexity, thereby reducing the overall sustainability and cost-effectiveness at an industrial scale [151]. 3D printing and atomic layer deposition (ALD) techniques are emerging for fabricating gradient membranes with controlled photocatalyst loading, thereby balancing activity and permeability [152]. However, these approaches remain largely experimental and face substantial barriers to commercial adoption. 3D printing of functional membranes is currently limited by material compatibility, production speed, and resolution constraints [153]. ALD, despite its exceptional precision, suffers from slow deposition rates, high equipment costs, and limited throughput [154]. As a result, their practical application in large-scale water-treatment membranes remains uncertain.

### 3.3. Reactor Designs and Configurations

**Figure 1:**
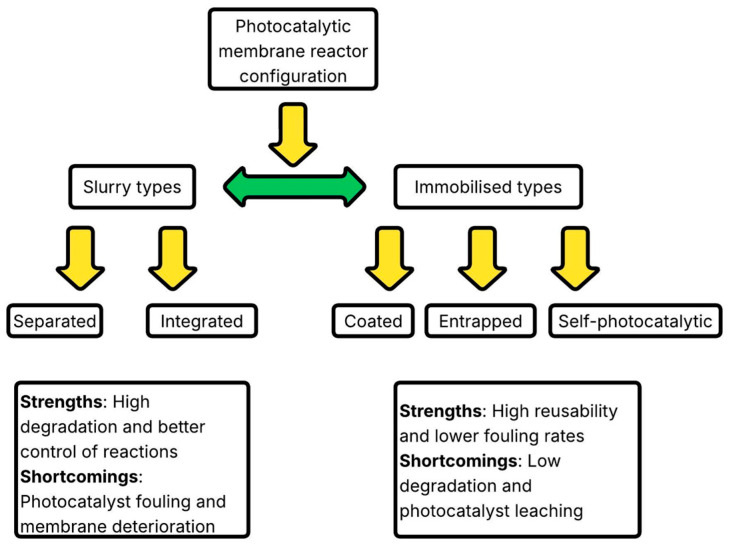
Classification of PMR configurations, their strengths, and shortcomings [78].

Photocatalytic membrane reactors are broadly categorised by the placement and mode of the photocatalyst: slurry or immobilised systems, as shown in Figure 1 [78].

**Figure 2:**
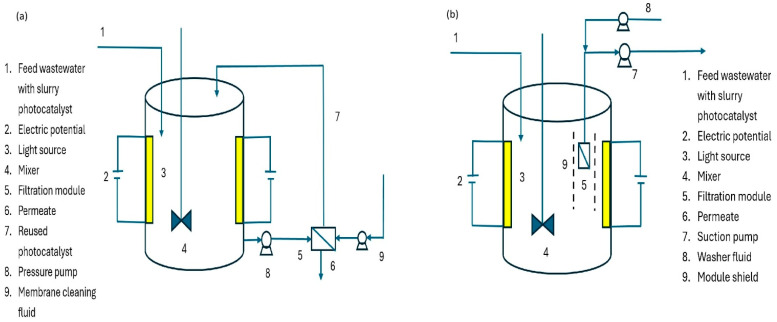
Slurry photocatalytic membranes: (**a**) separated type and (**b**) integrated types. Adapted with modifications from [157].

In slurry-type PMRs, photocatalyst particles are dispersed in the liquid phase, thereby providing a large surface area and direct contact with pollutants [40]. This configuration enhances degradation kinetics but poses challenges for catalyst recovery and may lead to secondary contamination. In comparison, previous studies report that slurry PMRs achieve higher degradation rates and a rejection rate of nearly 60% for dyes, but they exhibit higher energy consumption due to the need for constant agitation and catalyst separation [76,155,156]. Slurry systems can be classified as separated or integrated, as shown in Figure 2.

**Figure 3:**
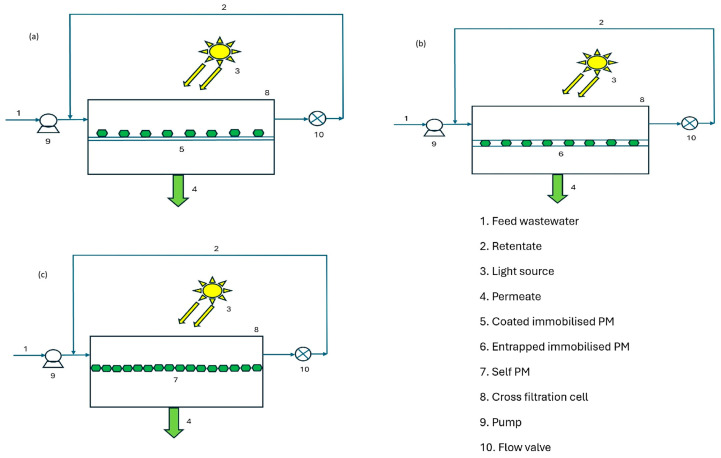
Immobilised PM: (**a**) coated, (**b**) entrapped, and (**c**) self, with photocatalyst indicated in green.

In separated types, photocatalytic reactions occur in one vessel, and the treated mixture is filtered in another. This reduces the membrane deterioration from UV and ROS [158]. The integrated slurry reactor, by contrast, combines photocatalysis and membrane filtration in a single module, enabling concurrent pollutant degradation and separation. The use of shielding baffles protects the membrane from UV-induced degradation while enabling efficient reaction control. However, nanoparticle-induced membrane fouling and higher transmembrane pressures remain major operational drawbacks [40]. From a cost and energy standpoint, slurry PMRs are suitable for high-throughput industrial effluents where catalyst recovery units can be economically justified [108]. For decentralised or small-scale wastewater treatment, the energy demand of stirring and separation often outweighs their benefits [159]. Immobilised PMRs feature the catalyst fixed to or embedded within a membrane, enabling simultaneous filtration and photocatalysis [82]. These can be classified as coated, entrapped, or self-photocatalytic membranes, as shown in Figure 3.

In coated membranes, the photocatalyst is immobilised on the membrane surface using techniques such as dip coating, electrospraying, or vacuum filtration [78]. This arrangement allows strong photocatalyst–pollutant interactions and enhanced mass transfer. The coating thickness must be optimised (typically 1–5 µm) to balance adhesion and light penetration, as excessive layers reduce photocatalytic activity [99]. In entrapped PMRs, the catalyst is embedded within the membrane matrix via electrospinning, wet spinning, or phase inversion. This configuration enhances the stability and minimises leaching but limits light exposure and reaction kinetics [160]. Emerging studies have integrated transparent polymer matrices with optical fibres to improve photon transmission and increase performance by up to 1.2-fold, mitigating internal shading effects [127]. Entrapped systems are ideal for long-term continuous operation when catalyst stability outweighs catalyst degradation. Self-PMRs are fabricated from inherently photoactive materials, such as TiO_2_ nanotubes or ZnO nanowires, and combine filtration and photocatalytic functionality [161]. They exhibit high surface area, strong hydrophilicity, and intrinsic antifouling behaviour. They often exhibit mechanical fragility and high synthesis costs due to complex anodisation and sintering steps [162]. Reinforcement through polymeric backing layers or ceramic supports can improve durability without compromising photocatalytic efficiency [163].

The choice of reactor configuration depends on the operational context: slurry reactors excel at rapid pollutant degradation in high-strength waste streams, while immobilised systems offer superior reusability and easier operation for low-strength or intermittent wastewater [76]. Future optimisation should focus on hybrid configurations, such as fluidised-bed PMRs and rotating-disk systems, that combine the high contact efficiency of slurry designs with the durability and reusability of immobilised membranes [164].

### 3.4. Factors and Considerations Influencing Performance of Photocatalytic Membranes

The performance of photocatalytic membranes (PMs) depends on a range of interrelated factors, including the mode of operation, photocatalyst immobilisation techniques, photocatalyst characteristics, light irradiation properties, feed water quality, and membrane material [165].

Each of these factors influences the contaminant degradation rate, membrane flux, fouling behaviour, and long-term operational stability. Recent research, emphasises that these parameters act synergistically, indicating that performance optimisation requires a multivariable approach rather than isolated adjustments [166]. The following subsections analyse each factor in detail, providing examples, recent data, and an evaluation of challenges and trade-offs.

#### 3.4.1. Mode of Operation

PMs may operate in dead-end or cross-flow configurations. In a dead-end operation, all feedwater flows perpendicularly through the membrane, leading to the accumulation of contaminants on the membrane surface and creating concentration polarisation [167]. This layer restricts mass transfer, lowers flux, and suppresses turbulence, thus reducing pollutant–photocatalyst interactions. A previous study reported that, in dead-end operation, degrading phenols reduced total organic carbon (TOC) by less than 10% [168]. This can be attributed to polarisation-induced shielding of the photocatalyst surface. The concentrated foulant layer further impedes photon penetration [169], thereby reducing the number of activated photoactive sites. Cross-flow operation recirculates the retentate, allowing shear forces to displace foulants and continuously minimise the thickness of the polarisation layer [24]. This results in higher light accessibility and degradation rates. The induced turbulence enhances both mass transfer and photocatalytic reaction kinetics, improving contaminant degradation and reducing fouling attachment [170]. Another study investigated the dead-end degradation of a 2,000,000 ng/L acid orange solution using TiO_2_ quartz fibre membranes under UV light. The performance was only 14%, indicating the shortcomings of dead-end photocatalytic degradation [171]. However, increasing transmembrane pressure to enhance flux can thicken the polarisation layer, reduce the residence time, and decrease the degradation efficiency [172]. Thus, optimal hydraulic pressure and flow velocity must be balanced to maintain efficient reaction–transport coupling.

#### 3.4.2. Photocatalyst Immobilisation Techniques

Photocatalyst immobilisation plays a critical role in determining the catalyst stability, light penetration, and mass transfer within photocatalytic membranes [76]. The immobilisation strategy should be clearly described and supported by morphological and structural characterisation, such as scanning electron microscopy, X-ray diffraction and X-ray photoelectron spectroscopy [173].

Surface immobilisation, such as dip coating, electrospraying, or vacuum filtration, provides direct exposure of the photocatalyst to incident light and contaminants [163]. However, variations in the coating thickness and adhesion influence leaching, optical, and mechanical durability. Excessive loading can induce pore blockage and light shielding [24], whereas insufficient loading limits reaction rates [98]; therefore, studies should justify the selected loading in terms of permeability, optical penetration, and long-term performance. A study investigated the effect of the thickness of the photocatalyst dip coating (0.26–21.9 µm) on alumina membranes. The optimal degradation thickness of methyl blue was 53% discolouration at 2.74 µm, whereas cracks in the coating were observed above 1.69 µm, leading to leaching and light scattering [174]. Surface modification techniques, such as silanisation or plasma treatment, can enhance photocatalyst anchoring by increasing the surface energy. Surface immobilisation is suitable for polishing applications such as municipal WWTPs’ tertiary effluent [175], where turbidity levels are low (less than 5 NTU), and particulate light-blocking is reduced, thereby maintaining better light access and reactivity. Tertiary effluents have low NOM loading, which is further advantageous, as the membrane is characterised by reduced competitive adsorption and fouling [176]. In long-term high-shear operation, the surface-immobilised membrane is unsuitable because surface adhesion relies on covalent bonding [177], which is not strong enough to resist detachment.

In-matrix immobilisation embeds photocatalyst particles within the membrane matrix, thereby improving mechanical stability, minimising leaching, and reducing photon accessibility and radical formation due to internal light scattering [82]. They are suitable for secondary municipal WWTPs effluents (3–10 mg DOC/L) where NOM and turbidity levels are moderate (5–50 NTU and 3–10 mg DOC/L), as it maintains its photoactivity due to in-matrix protection and relies on pore-level photocatalyst pollutant interactions. The in-matrix protection of the photocatalyst reduces NOM adsorption, thereby reducing photocatalyst poisoning and maintaining photocatalytic activity. Reduced leaching increases the capability of the in-matrix immobilised membrane to be utilised in moderate-shear operations, such as cross-flow filtration [178]. A recent study employed improved dispersion of photocatalysts within mixed-matrix membranes to enhance light propagation. The photocatalytic membrane outperformed surface-distributed photocatalysts on the membrane, degrading phenols by 96%, 3% higher, while using 39 W/cm^2^ less power [174].

Self-PMs consist entirely of photocatalytic materials such as TiO_2_ nanotubes or ZnO nanowires [178]. These membranes possess extensive photoactive surfaces but are mechanically fragile and require composite reinforcement. Their production involves advanced techniques such as anodisation or electrospinning, which increase the fabrication costs [179]. To address this, hybrid polymer–ceramic scaffolds are being explored to achieve higher mechanical stability and performance. A study used TiO_2_ structures supported on a Pluronic block copolymer and achieved superior mechanical interconnectivity, with over 40% dye degradation and 20% less flux decline than a TiO_2_ ceramic membrane [180]. Self-PMs are suitable for raw wastewater characterised by high turbidity (above 50 NTU) and high NOM, as reactivity and antifouling are the priorities [24]. Improvements in mechanical properties make them ideal for elevated-shear applications with reduced leaching.

Photocatalyst loading must be reported in quantitative and reproducible units, such as wt% relative to membrane mass or surface loading (ng cm^−2^) [181]. Each immobilisation method involves trade-offs among photocatalytic efficiency, durability, and scalability when selecting a suitable membrane type, as shown in Table 5. For large-scale operations, dip-coated or spray-coated surfaces remain the most feasible due to their simplicity, controllable uniformity, and cost-effectiveness [182,183].

#### 3.4.3. Photocatalyst Characteristics

The physicochemical properties of photocatalysts, such as the bandgap, particle size, point of zero charge (PZC), crystallographic phase, and stability, directly determine the photocatalytic membrane performance [184]. The bandgap dictates the photon energy required to generate electron–hole pairs. Narrow-bandgap semiconductors such as Fe_2_O_3_ and CdS can utilise visible light, whereas wide-bandgap materials such as TiO_2_ require UV light. TiO_2_ (3.2 eV) absorbs wavelengths of less than 387 nm, while modified BiVO_4_ (2.4 eV) extends absorption up to 520 nm [185,186]. Advanced doping and heterojunction engineering have reduced the charge recombination and broadened the spectral response of photocatalysts, improving the efficiency [78].

The PZC influences adsorption behaviour. Below the PZC, photocatalysts are positively charged; above it, they become negatively charged [187]. This charge behaviour dictates electrostatic interactions with pollutants. ZnO with a PZC of 9.1 efficiently degrades anionic dyes in acidic media, such as methyl orange (90% removal at pH 6), compared to cationic dyes, such as crystal violet (80% removal at pH 4), while TiO_2_ with a PZC of 6.3 is more effective for cationic species under neutral to basic conditions [188,189].

A smaller particle size increases the surface area and the number of active sites, thereby improving the degradation rates [190]. However, too-small nanoparticles (e.g., those less than 10 nm) are prone to agglomeration or leaching upon immobilisation. For coated photocatalytic membranes, a reduced particle size enhances the long-term flux but may slightly decrease the permeability [16].

The crystallographic phase strongly affects the photoactivity. Anatase TiO_2_ is typically more photoactive than rutile or brookite [191] and therefore would result in more reactive photocatalytic membranes when immobilised [147]. Similarly, hematite is the most photoactive phase of Fe_2_O_3_ [192]. Mixed-phase photocatalysts, such as anatase–rutile TiO_2_ composites, exhibit synergistic charge transfer and achieve up to 50% higher degradation efficiency than single-phase materials. A previous study reported a reaction rate constant of 0.024/min for mixed-phase titanium dioxide, whereas rutile and anatase TiO_2_ had rate constants of 0.01/min for the degradation of the methyl blue dye [193].

The stability and toxicity of photocatalysts determine their environmental viability. Titanium dioxide remains the preferred choice due to its low toxicity and resistance to photochemical corrosion [194] when used to synthesise photocatalytic membranes. Novel visible-light photocatalysts, such as Cu_2_O or Ag_3_PO_4_, are prone to photocorrosion, leading to leaching and necessitating surface passivation strategies [173] when immobilised in or on membrane surfaces.

#### 3.4.4. Light Irradiation Properties

Two familiar light sources are UVC and visible light [195]. Visible-light-driven systems are increasingly favoured because visible light accounts for 45% of solar irradiance, whereas UV accounts for only 5% [196]. The lamp emission mode also affects the performance. Pulsed irradiation enhances charge-separation efficiency compared to continuous irradiation by reducing recombination. In a previous study, the frequency of a 365 nm lamp was reduced below 0.8 Hz, resulting in toluene deposition of 0.16 cm/s [197]. The intensity of light influences the rate of radical formation. At low intensities below 20 W/m^2^, radical generation is directly proportional to the light intensity, whereas above 25 W/m^2^, recombination dominates [198,199]. By contrast, a previous study used TiO_2_ immobilised on alumina membranes to degrade methyl blue dye. The study identified an optimal discolouration of 87% at 350 W/m^2^ [200]. Advanced photoreactor geometries, such as annular or tubular designs, can improve photon utilisation efficiency and enhance degradation by 2.2-fold compared to slurry systems [201]. Studies should therefore include the light source type and manufacturer, spectral range and peak wavelength, irradiance at the membrane surface (W/m^2^ or mW/cm^2^), distance between the light source and the membrane, and reactor geometry. Where possible, photon flux or apparent quantum yield should be provided to allow cross-study normalisation. Importantly, the optical path length and matrix light attenuation should be discussed, particularly for turbid or coloured wastewaters, where inner-filter effects significantly reduce effective irradiance.

#### 3.4.5. Feed Water Characteristics

Feed water properties, including the contaminant load, pH, temperature, inorganic ions, and dissolved oxygen (DO), significantly influence photocatalytic membrane degradation performance [202].

Typically, CEC degradation follows pseudo-first-order kinetics at low pollutant concentrations [203]. As the organic load increases, the degradation initially rises until the availability of photogenerated radicals becomes the limiting factor, after which the efficiency declines. A study reported an optimal tetracycline contaminant range of 10,000,000–20,000,000 ng/L for stable photocatalytic performance; beyond 50,000,000 ng/L, self-shading and radical competition reduce the degradation rates [105].

The pH affects both the photocatalyst’s charge and contaminant ionisation [204]. A neutral pH is generally optimal for radical formation [205,206]. However, for weakly ionised pharmaceuticals such as sulphamethoxazole, slightly acidic conditions enhance adsorption-driven degradation. A previous study reported the highest degradation of sulphamethoxazole (81%) and a relative flux of 0.78 at pH 4 [16,166].

Temperature influences both adsorption and charge separation efficiency. Low temperatures reduce contaminant adsorption, whereas temperatures above 80 °C promote charge recombination. Optimal photocatalytic activity typically occurs between 20 °C and 80 °C [178,184]. Recent work confirms an Arrhenius-type relationship between temperature and reaction rate constant up to 70 °C, beyond which recombination predominates [207,208].

Inorganic ions such as Cl^−^, NO_3_^−^, SO_4_^2−^, and CO_3_^2−^ act as radical scavengers, influencing degradation rates in different ways [209,210,211]. For example, doubling the NaCl concentration reduced the TOC removal of sulphametoxazole from 58% to 46%, as chloride anions suppressed the hydroxyl radical availability [166]. Additionally, a previous study quantified the effect of competing ions on diazinon degradation. The reaction constants were 0.0277/min with SO_4_^2−^, 0.0234/min with NO_3_^−^, and 0.0166/min with Cl^−^ [212]. In contrast, oxidising ions such as S_2_O_8_^2−^ and IO_3_^−^ enhance degradation via electron scavenging [213]. While H_2_O_2_ addition improves degradation, excessive dosages quench radicals [214]. Dissolved oxygen acts as an electron acceptor, suppressing recombination and promoting superoxide radical formation [215].

Real wastewater contains diverse organic and inorganic matter that rapidly accumulates on membrane surfaces, reducing flux and light penetration [216]. Previous studies have shown that, without mitigation, fouling can reduce flux by up to 50% within 48 h [132]. Solutions include periodic backwashing, air scouring, ultrasonic cleaning, and intensified photocatalytic self-cleaning via higher UV flux [217,218]. Operating at slightly lower flux or increasing effective membrane surface area also minimises foulant deposition. The aeration intensity should be optimised since excessive bubbling causes light scattering and reduces adequate illumination [40]. A trade-off arises when using real wastewater matrices: NOM competes for radicals, lowering degradation efficiency but mitigating membrane fouling. Hence, pretreatments are increasingly recommended to ensure stable operation in full-scale wastewater treatment [219].

Studies should therefore report the pH, conductivity, turbidity, dissolved organic carbon (DOC), total dissolved solids (TDS), and the concentrations of major inorganic ions. The type and origin of the matrix synthetic feed (municipal or industrial wastewater) must be clearly specified, as the performance obtained in synthetic wastewater is not representative of real wastewater.

#### 3.4.6. Membrane Material

The membrane material plays a key role in enhancing the synergistic performance of photocatalytic membranes by providing mechanical support for the photocatalysts. It increases HRT, allowing increased photocatalytic degradation of CECs and their reaction intermediates. The membrane substrate dictates mass-transfer properties, surface chemistry, and photodegradation resistance [40]. Photocatalytic membranes synthesised from polymeric materials are prone to UV-induced chain scission and radical attack, leading to the loss of selectivity [220]. Their organic nature also promotes fouling. PVDF membranes, however, offer improved chemical stability, UV and radical resistance and are relatively low cost [200]. Surface modification to enhance photocatalyst adhesion and hydrophilicity, in turn, enhances pollutant adsorption and degradation [17]. A previous study increased the amount of graphene oxide incorporated into the PVDF membrane by 0.3%. The membrane reduced its chemical deterioration by 5% [221]. Ceramic photocatalytic membranes, in contrast, are thermally and chemically robust, offering higher flux and hydrophilicity. A previous study reported up to 100% flux recovery after backwashing and solar irradiation using zinc-coated ceramic photocatalytic membranes [222]. However, ceramic materials are more expensive and brittle, thereby limiting their use in large, flexible systems. Hybrid polymer–ceramic membranes provide a middle ground, achieving cost reduction relative to full ceramics while maintaining comparable flux [91].

## 4. Application of Photocatalytic Membranes

Photocatalytic membranes have demonstrated high contaminant-removal capability in a controlled laboratory environment. To translate this into an industrial application, intermediate-scale pilot studies are conducted. The technological readiness level of photocatalytic membranes is analysed and discussed.

### 4.1. Scale-Up Systems

Lab-scale PMs have demonstrated the effective removal of CECs under controlled conditions of key performance factors [78]. Scaling up to pilot systems introduces challenges that cannot be directly inferred from laboratory results due to differences in hydrodynamics, increased fouling behaviour, complex light distribution, and increased operational volume [222]. Lab-scale systems typically operate at a few litres per hour with idealised synthetic feed, allowing close monitoring of fouling, pH, and light intensity. In contrast, pilot systems process tens to hundreds of litres per hour, often using real wastewater matrices with varying turbidity, ionic strength, and pollutant load. These conditions result in unpredictable fouling patterns, uneven illumination, and variable photocatalyst efficiency [223]. Scaling up can increase photocatalyst leaching, membrane stress, and pressure drops that are negligible in bench-scale tests [95]. A pilot-scale study of a slurry photocatalytic membrane observed a 4 kPa increase in suction pressure within 40 min, attributed to photocatalyst blockage of the membrane during dye degradation [224]. Pilot studies facilitate the evaluation of regulatory compliance and safety, including UV exposure risks, photocatalyst toxicity, secondary pollutant formation, and compliance with local wastewater discharge standards [225]. Environmental regulations such as Commission Regulation (EU) 2018/1881 often require the monitoring of photocatalyst leaching, residual oxidants, and ROS byproducts in permeate streams to prevent secondary contamination [136].

### 4.2. Requirements Considerations of Scaling Up

The scale-up process begins with lab validation, assessing the photocatalyst selection, membrane type, and pollutant degradation efficiency [226]. Once laboratory efficacy is confirmed, a prototype system is designed with careful attention to geometric similarity, hydrodynamics, and modularity, often assisted by computational simulations to predict larger-scale performance [227]. Scaling up requires maintaining adequate HRT and optimising the pump, pipe, and control systems to handle higher volumetric flow while minimising the labour intensity [170]. Dynamic automation using programmable logic controllers (PLC) and integrated sensors for level, pressure, and flow ensures real-time control and consistent light delivery across larger membranes [228].

Economic feasibility is a core consideration. Capital expenditure (CAPEX) includes membrane modules, reaction chambers, UV or visible-light lamps, light concentrators, pumps, piping, tanks, and control units, while operating expenditure (OPEX) includes pumping, lighting energy, membrane cleaning, oxidant dosing, labour, and waste disposal [229]. Although complete techno-economic analyses may be beyond the scope of early-stage studies, minimum economic reporting should include energy consumption (lighting and pumping), material and fabrication costs, and an estimate of membrane lifetime or the number of reuse cycles. Costs should be normalised to treated wastewater volume (e.g., cost per m^3^) wherever possible, and key scale-up assumptions should be clearly stated [170].

Regulatory and safety compliance with legal requirements, such as the Commission Implementing Decision (EU) 2025/439, is critical, as it establishes a new watchlist of contaminants suspected of posing risks to the ecosystem and human health, which limits photocatalytic membrane discharge, including intermediates [230]. Pilot-scale systems must therefore prevent human exposure to UV radiation and reaction intermediates, minimise photocatalyst leakage, and withstand environmental conditions when operated outdoors. Protective barriers, automated UV shielding, and photocatalyst immobilisation strategies are increasingly applied to meet these requirements [135]. Table 6 summarises several applied, scaled-up PM systems, including the membrane type, capacity, removal efficiency, operational costs, and specific water matrices.

As shown in Table 6, high-volume slurry systems, such as Ce–Y–ZrO_2_/TiO_2_ for treating industrial laundry wastewater, achieve near-complete turbidity and microplastic removal (99.9%) at low OPEX (0.93 €/m^3^) due to efficient UV-photocatalytic reactions and high photocatalyst retention [231]. Immobilised systems such as PS-TiO_2_-NF with persulfate achieve moderate contaminant removal of 62% at higher operational costs (572.32 €/m^3^) due to slower degradation kinetics and additional oxidant consumption [225]. Smaller-capacity systems (TiO_2_/PVDF hollow fibres and TiO_2_/MF) demonstrate variable pesticide and DOC/BOD removal, indicating that the performance depends strongly on the feedwater complexity, membrane configuration, and light availability [219,227]. Therefore, the system design must balance capacity, efficiency, and cost while accounting for feedwater complexity and available illumination. Pilot systems cannot simply replicate lab-scale conditions; adaptation for fouling management, light distribution, and photocatalyst retention is essential [135].

The current pilot-scale photocatalytic membranes rely on high-energy-consuming UV lamps and pristine TiO_2_ as photocatalysts. This lowers the viability, as it uses only UV wavelengths and requires additional oxidants to increase the efficiency [225]. More effort is needed to investigate less energy-intensive photocatalysts through modifications such as doping, dye sensitisation, and heterojunction formation. This will reduce the dependence on UV lamps and oxidants.

### 4.3. Technological Readiness Level (TRL)

**Figure 4:**
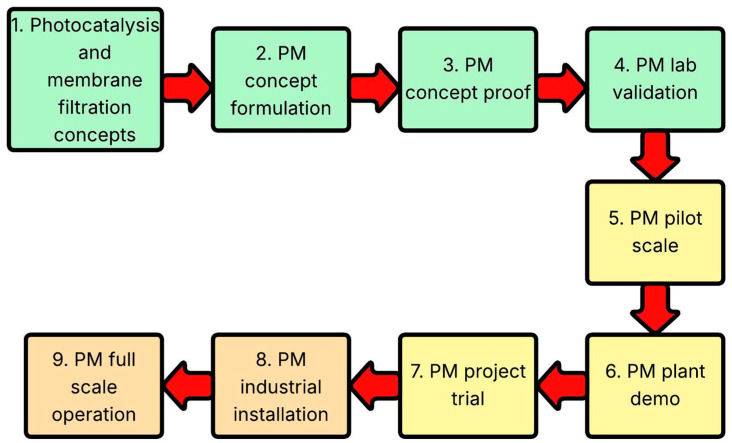
Technological readiness level as adapted to PM.

TRL is a scale used to analyse the level of application from the inception of ideas to the commercial application [98]. TRL levels are divided into nine core stages (Figure 4), with three major phases: the research phase (levels 1–4), indicated in green; the scale-up phase (levels 5–7), displayed in yellow; and the industrial adoption phase (levels 8 and 9), indicated in orange [232].

These are applied to photocatalytic membranes as an essential tool for identifying challenges and requirements, and they provide a clear roadmap for guiding researchers. Stage 1 involves identifying basic concepts, such as photocatalysis and membrane filtration, as single entities. Stage 2 involves representing the ideas for a photocatalytic membrane that combines photocatalysis and membrane filtration [24]. The third stage involves investigating concepts, where most studies utilise semiconductors in conjunction with membranes, either in slurry or immobilised form [233]. The fourth stage is where concepts are validated in labs. Examples of such studies include [16,234,235,236]. This is the current state of research, showing how photocatalytic membranes can be utilised to remove contaminants such as CECs. This period involves developments aimed at improving and optimising the performance of photocatalytic membranes [237]. The fifth stage will include pilot scaling, demonstration, and a full-scale trial in the region shaded yellow. This will be the next phase, investigating pilot-scale operations, costing, and performance at larger scales [238]. Examples of these studies include [225,227,231]. The TRL tool identifies the next phase as pilot-scale demonstration and trial, located between lab-scale studies and commercialisation, as indicated in the yellow region. Previous research agrees closely with the current TRL stage and indicates that the final phase can involve industrial application and full-scale operation [98].

## 5. Conclusions and Future Perspectives

The increasing occurrence of CECs in surface and groundwater underscores the limited effectiveness of conventional treatment processes in removing these compounds, thereby posing ecological and human health risks. Photocatalytic membranes offer a promising solution by synergistically combining membrane separation with photocatalytic degradation. They can mineralise CECs into harmless products, such as carbon dioxide and water, thereby reducing their persistence and toxicity. It is important to emphasise that the actual value of photocatalytic membranes lies not only in their contaminant removal but also in their potential to transform conventional treatment by integrating degradation, separation, and self-cleaning in a single system.

A more critical assessment reveals that despite significant advances in photocatalyst development, such as visible-light-responsive TiO_2_, heterojunction-based materials, and membrane immobilisation techniques, key limitations remain. These include catalyst leaching, inconsistent light distribution in scaled systems, reduced photonic efficiency due to membrane opacity, and the lack of robust, cost-effective, and industrially scalable fabrication routes. Notably, most reported successes still originate from highly controlled lab studies using synthetic matrices, which do not capture the complexity of real wastewater. These limitations directly affect the technology’s long-term stability, cost-effectiveness, and regulatory acceptance.

Scaled-up applications demonstrate the feasibility of photocatalytic membranes. Yet, operational setbacks, including accelerated fouling, increased energy demand for illumination, photocatalyst detachment, and lower fluxes under real-world feed conditions, continue to hinder practical deployment. Addressing these constraints will be critical for advancing from promising prototypes to fully operational pilot and demonstration systems.

The following recommendations are proposed: (i) Future research should provide actionable implementation-oriented strategies including adopting low-cost immobilisation methods such as spray deposition with chemical anchoring, transitioning from energy-intensive UV lamps to passive solar concentrators, integrating daylight-driven catalysts that reduce dependence on external electricity, and designing modular reactors that can be incorporated into existing treatment units without extensive capital investment. (ii) To improve technical performance, studies should prioritise the development of mechanically robust membranes with strong photocatalyst anchoring, such as covalent grafting, crosslinking, or MOF-based interlayers, to minimise leaching and enhance operational stability. Transparent or semi-transparent membrane supports should be explored to overcome current constraints on light penetration. (iii) More comprehensive characterisation of real wastewater fouling mechanisms, inorganic interference, and catalyst deactivation pathways is required. Understanding these limitations will guide operational optimisation, including pre-treatment requirements, hydrodynamic modifications, and adaptive cleaning strategies. (iv) The identification and toxicity assessment of degradation intermediates should be integrated into every photocatalytic membrane study. Because some intermediates are more toxic than the parent CECs, future work should integrate analytical monitoring with risk-based assessments to determine safe hydraulic retention times and optimal illumination conditions. (v) Large-scale pilot studies must be expanded, with transparent reporting on performance decline, operational costs, light-distribution challenges, and membrane service life. Without these data, techno-economic assessments remain speculative and cannot inform realistic pathways to commercialisation.

## Figures and Tables

**Table 1:** Classification of selected contaminants of emerging concern, examples, wastewater treatment effluent concentrations, and their effects.

Class	CECs	WWTPs Effluent Range	Effects	References
Hormonal steroid	17R-ethinyl estradiol (EE2), estrone (E1), 17β-estradiol (E2), estriol, isoflavoids, lignans	0.01–265 ng/L	Feminisation of male fish, population decline, change in reproductive behaviour, and hormonal imbalance	[59]
Antibiotics	Sulfamethoxazole, sulfadimethoxine, ciprofloxacin, norfloxacin, azithromycin, erythromycin, tetracycline, amoxicillin, and trimethoprim	4.7–6840 ng/L	Antibiotic resistance, genotoxicity, delayed growth, and algal growth	[60,61]
Anti-inflammatory drugs	Acetaminophen, ibuprofen, and naproxen	10–50 ng/L	Neurotoxicity, reproductive impairment, and oxidative stress	[61,62]
Surfactants	Alkylphenols (APs), alkylphenol ethoxylates (APEOs), and alkylphenol carboxylates (APECs)	570–751,000 ng/L	Endocrine disruption, persistence, and bioaccumulation	[61,63]
Preservatives	Parabens	1630–12,200 ng/L	Persistency, bioaccumulation, reduced fertility, and carcinogenicity	[64,65]
Pesticides	Triazine herbicides, chlorophenoxy herbicides (CPHs), organochlorine insecticides (dichlorophenol derivatives, e.g., 2,4-D, pentachlorophenol (PCP), DDT, hexachlorocyclohexane, or lindane)	852–82,044 ng/L	Carcinogenicity, neurotoxicity, persistence, and bioaccumulation	[66]
Industrial chemicals	Phthalate, bisphenol A (BPA), polycyclic aromatic compounds, polyaromatic hydrocarbons (PAHs), polychlorinated dibenzodioxins (PCDDs) or dioxin, and polychlorinated dibenzofurans (PCDFs), polychlorinated biphenyls (PCBs), brominated flame retardants [e.g., polybrominated biphenyls (PBBs), and polybrominated diphenyl ethers (PBDEs)]	13,400–107,000 ng/L	Metabolic disorder, immunotoxicity, and neurotoxicity	[67,68]

**Table 2:** Total organic carbon (TOC) removal efficiencies for sulfamethoxazole (SMX) using various advanced oxidation processes (AOPs).

Methods	Pollutant Concentration (ng/L SMX × 10^7^)	pH	Treatment Duration (min)	TOC Removal (%)	Reference
Ozonation	2	5.2	60	16	[73]
Fenton/goethite	1	7	70	40.2	[74]
ZnO Photocatalysis	1	4	360	20.8	[75]
Photocatalytic membrane (NTiO_2_/PVDF)	1	7	100	65	[16]

**Table 3:** Photocatalytic membrane grafting techniques with their strengths and weaknesses.

Grafting Techniques	Strengths	Weakness	Reference
UV-induced	High grafting rates and strong immobilisation of the photocatalyst	Require specialised equipment and may cause polymer damage	[83]
Plasma-induced	Rapid, uniform and high wettability	Weak adhesion and effective for shallow surfaces	[84]
Radiation-induced	High stability, uniformity and customised grafting levels		[85]
Chemical-induced	High wettability and enhanced selectivity	Potential residual pollution	[86,87]
Polydopamine-induced	Enhance stability and increased photoactivity	Slow deposition kinetics and pH-sensitive	[88]

**Table 4:** The effects of catalyst loading on permeability for the photocatalytic membranes.

Optimum Photocatalyst Loading	Type of Membrane	Observation	References
13–15 µm 3%TiO_2_ and polyvinyl alcohol (PVA) coating	PVA-coated polyvinylidene fluoride (PVDF) membrane	Reduced porosity from 37.6% to 32.5% beyond the optimum coat	[99]
1% TiO_2_ weight loading	Mixed matrix PVDF	Reduced permeability above 2% loading	[100]
2 × 10^9^ ng/L TiO_2_ slurry	TiO_2_ ceramic	Stagnating flux level beyond 2% loading	[101]

**Table 5:** Trade-offs in selecting a suitable membrane type for a given membrane property.

Property	Surface Immobilised Membranes	In-Matrix Immobilised Membranes	Self-Photocatalytic Membranes
Turbidity	◊	◊ ◊	◊ ◊ ◊
NOM levels	◊	◊ ◊	◊ ◊ ◊
High shear or frequent backwashing	◊	◊ ◊	◊ ◊ ◊
Leaching risk	◊ ◊ ◊	◊ ◊	◊
Fabrication ease/Scaling up	◊ ◊ ◊	◊ ◊	◊

Potential key: high ◊ ◊ ◊, moderate ◊ ◊, and low ◊.

**Table 6:** Applied scaled-up photocatalytic membranes, indicating performance and costs in Euros.

Membrane System	Capacity	Targeted CECs	Efficiency	Cost (OPEX)	References
Ce–Y–ZrO_2_/TiO_2_, slurry, UV powered and industrial laundry wastewater	72 m^3^/day	Microplastics	99.9% removal of turbidity, colour, suspended solids, and microplastics	0.93 €/m^3^	[231]
PS-TiO_2_-NF, immobilised, persulphate, UVA, and dye water	0.519 m^3^/h	Brilliant blue FCF dye	62% removal	572.32 €/m^3^	[225]
TiO_2_/PVDF hollow fibres, UV, and agrowastewater	1.2 m^3^/day	Acetamiprid and thiabendazole	25–41.5% pesticide removal		[227]
TiO_2_/MF, H_2_O_2_, UV, solar, and real wastewater	1.2 m^3^/day	Acetaminophen, amoxicillin, erythromycin, tetracycline, sulfamethoxazole, carbamazepine, chloroquine, diclofenac, ketoprofen, naproxen, haloperidol, and trazodone	62.5% DOC and BOD removal (meets EU 2020/741 requirements)	4.79 €/m^3^ before optimisation and 2.60 €/m^3^ after optimisation	[219]

## Data Availability

The original contributions presented in this study are included in the article. Further inquiries can be directed to the corresponding author(s).
